# Medical Organisation and the Growth of the Medical Sciences in the Seventeenth Century, Illustrated by the Lives of Local Worthies
1Presidential Address to the Bath and Bristol Branch of the British Medical Association, delivered at the Annual Meeting, held in Bristol, June 14th, 1911.


**Published:** 1911-09

**Authors:** George Parker

**Affiliations:** Physician, Bristol General Hospital; Lecturer on Medical Jurisprudence, University of Bristol


### The Bristol Medico-Chirurgical Journal.

*" Scire est nescire, nisi id me*
*Scire alius sciret."*

SEPTEMBER, 1911.

### MEDICAL ORGANISATION AND THE GROWTH OF THE MEDICAL SCIENCES IN THE SEVENTEENTH CENTURY, ILLUSTRATED BY THE LIVES OF LOCAL WORTHIES.[1]

BY

GEORGE PARKER, M.A., M.D. Cantab.
*Physician, Bristol General Hospital;*
*Lecturer on Medical Jurisprudence, University of Bristol.*

I AM sincerely grateful for the honour you have done me in electing me to this office, an honour which I appreciate the more when I think what great and notable men have held this chair in the past. I can only endeavour to do my best as a faithful servant of the Branch in the coming year.

I do not intend to-night to discuss the progress which has been made in the knowledge of medicine since I was a student. Nor shall I ask you to listen to a political discussion on the anxious problems of the great struggle in which we are now

[1] Presidential Address to the Bath and Bristol Branch of the British Medical Association, delivered at the Annual Meeting, held in Bristol, June 14th, 1911.

involved for our means of living and for our professional status, a struggle the results of which we cannot foresee, and about which we only know one thing, that we are determined to fight to the end and to win.

No, to-night I thought it would be a relief to turn to an old-world picture, and to ask you to consider with me the medical organisation, and the growth of the medical sciences in the seventeenth century, as illustrated by the lives of our local worthies. It was a century of the greatest importance in the history of medicine on account of the brilliant development of natural science and medicine in England at the time. In certain respects it is nearer to us than the eighteenth century from the extraordinary interest then felt in scientific subjects, the use of the inductive method of study, and the rapid growth of the physical sciences, which then, as now, gave us more facts than could be utilised at once in the healing art. The acquisitions of physiology were, perhaps, actually greater than those of the nineteenth century, for in a brief space of time the main facts of the circulation, digestion, respiration and secretion were discovered. The world had to wait for a hundred years before great additions were made. On the other hand, the organisation of medical practice was singularly unprogressive, and provision for public health was barbarous.

Let us take this side of our subject first. The age was not much behind our own in the joys and profits of quackery, but there were many highly-qualified men, and to protect the public certain corporations were authorised to grant licences to practise after examination. These were (1) the Universities ; (2) diocesan officials ; (3) certain great guild companies in London, Edinburgh, Bristol and other cities ; and (4) the Royal College of Physicians. Again, we must remember that there were four classes of doctors—physicians, apothecaries, pure surgeons and barber-surgeons. Mr. Harsant,[1] speaking of a little later date, tells us that there were in Bristol five physicians, nineteen surgeons, thirteen barber-surgeons and twenty-nine apothecaries.

[1] *Bristol M.-Chir. J.*, 1899, xvii, 297.

*The Physicians* were originally University graduates, scholars indeed, yet badly trained in their profession. During the later Renaissance they had gained the benefits of organisation. For Linacre, after studying in Italy, rose into high favour with Henry VIII, and representing to him the enormous advance of medicine abroad, pleaded for a great English foundation, and the Royal College of Physicians was the result in 1518. Linacre's belief in the new learning and Greek medicine led him to restrict his college in some degree to men from Universities, and ever since its members have been distinguished for their literary attainments. I shall speak later on of certain Bristol physicians in this century who helped in the development of medicine; but others must be commemorated, such as John Counsell, Jeremy Martin, John Deighton, Richard Baskerville, William Gibbes (physician to Henrietta Maria) and Charles Thirlby. In the troublous times of the Civil War ministers of religion, when their party was defeated, often studied again and qualified as doctors. Thus we find practising at Bristol Francis Cross, an ex-Fellow of Wadham, and Ichabod and Robert Chauncey, sons of the first President of Harvard. Ichabod was for a time a Parliamentary Army Chaplain, and while practising medicine here later on was banished the country in 1684.

*The apothecaries* were at first simply grocers dealing in drugs, and subject to the inspection of the College of Physicians. In London James I formed them into a separate company in 1624, but I can trace no organisation of them whatever in Bristol. About the middle of the century they found an opening for practice in applying the fashionable blisters and enemata. They soon grasped much of the physicians' visiting work, merely calling in a physician when all else failed. As they could not legally charge for advice before 1858, and as the fashion for drugs increased they were led into inflicting on the sick immense quantities of medicines, each dose often put up in a separate bottle with a long white label. Like other craftsmen, they served a seven years' apprenticeship, and could then get the freedom of the city and the right to practise in it, subject originally to inspection by physicians and diocesan officials. Many of the Bristol apothecaries were men of considerable standing. Day, Harris, and Dale were sheriffs, Millecheape helped to analyse the Hot Well waters, and in later days the wealthiest general practitioners were apothecaries.

*The barber-surgeons'* story will be best understood if I take first their history in London. This curious combination of callings is often said to be due to a law of the Church in 1163 forbidding priests to practise surgery, but as she also often forbade them neglecting their work to gain wealth as physicians the story is doubtful. It is probable that barber-surgeons arose first as the keepers of medicinal bathing-houses, or as we should say private sanatoriums, which were in great favour at one part of the Middle Ages. These bath-keepers naturally took up some sort of massage, bleeding and dental work as well as shaving and perfuming their clients. In London the barber-surgeons existed before the time of Edward II, and their society became one of the city companies with a share in the government of London They obtained a charter from Edward IV, and the power of examining and inspecting surgeons. Even then there was a distinction between the members practising surgery and those practising barbery. Now side by side with this company was a small guild of pure surgeons which had grown up during the Hundred Years' War, "men of the long robe," as they were called. Their society, too, had obtained some official recognition, and on the whole worked harmoniously with the barber-surgeons. At last, in 1540, an Act of Parliament united the guild and the city company, and gave them full powers of licensing surgeons in and around London. They rose to the occasion and instituted (1) weekly lectures on surgery, some of which have continued to the present time, together with (2) examinations and (3) dissections, ordering that members which practised one craft should abstain from the other. This curious matrimonial union of surgeons and barbers went on till 1745, and did very good work until the surgeons seceded, formed again a society of their own, and finally got a charter for the present College of Surgeons in 1800.

Now in Bristol we find an imitation of the London system, but as the Company of Barber-Surgeons here was not strengthened by an Act of Parliament they had only the powers of a mediæval corporation, and when the companies fell into disrepute in the eighteenth century for restricting trade they shared in the general destruction.

The earliest mention of them is in the *Little Red Book of Bristol,* where they appear as barbers pure and simple. In 1395 the municipal authorities, with the consent of the Company, closed their shops on Sundays, except for strangers suddenly coming into the city, and on six Sundays in the autumn, and on Christmas Eve if that fell on a Sunday. In 1418 it was laid down that since ignorant people, such as carters and smiths, daily carry on this craft, no one henceforth may poll or shave except members of this guild, provided that a man's own servant may poll or shave him in his house, and that two servants dwelling together may poll or shave each other. No apprentice may be taken for less than seven years.

Again in 1439 the mayor, sheriffs and goodmen of the city confirm the rules of the Guild Company, and impose fines on offenders. Hence it had a clear legal status for the future.

Then follows a long blank in their history. Mr. Francis Fox, however, has kindly allowed me to see some MSS. in his possession from which important facts are to be learnt. First it is clear that at some unknown date surgeons were definitely included as in London, and apparently provision was made for their instruction. At any rate, in 1652 the Common Council of the city declared that whereas "the Company of Barbers and Chirurgeons" had been anciently incorporated, though some of their charters were even then illegible, they shall be established and ratified, and have power to make ordinances, and to buy and hold lands. No one shall be allowed to exercise the craft unless he be free of the city. As to the barbers, they were forbidden to carry on their trade on the Sabbath; and as to the surgeons, no one of the Company or city shall take in hand a cure until he be allowed by the Company to be a sufficient workman, skilful in the art of surgery, and made free of the said Company, except in a time of extremity when no chirurgeon can be found, or it be done gratis to a poor body.

No chirurgeon shall take a case out of another's hand or before such time as the first holder of the case be satisfied and contented for his pains, *i.e.* his bill is paid. The same to be judged by two persons. All persons of the Company who take in hand a cure shall do their uttermost, and give due attendance to the best of their power and skill until the cure is ended, under a penalty of £5.

No person shall undertake to cure a wound or grief which is in danger to lose life or limb before he acquaint two or three of the chiefest and most skilful therewith, who may view the same cure before he take it in hand.

If any poor or impotent person of the city shall have cause to use the help of a chirurgeon, the master shall appoint a sufficient man to cure him. After the cure is ended the chirurgeon shall send in a true bill of charges, and the mayor and two aldermen shall determine what the person shall pay for salve and medicine. The chirurgeon to do such a cure gratis if the person be unable to pay for the same.

Every surgeon entering for a voyage as surgeon of a ship of the port shall have his chest examined by the master and two others, that it be well furnished with all things necessary for the said voyage.

In 1672 another ordinance is found in the MSS. regulating the number of apprentices.

Thus we see that there was at this time a powerful society governing the practice of surgery in Bristol. It is commonly said that their Guild Hall has vanished, but only this week, by the help of Mr. John Pritchard, I discovered it still existing in a dismantled condition, and forming the upper part of the Bunch of Grapes Inn in Exchange Avenue. The great hall for meetings was probably on the first floor, and over part of an old timbered room on the second floor a glazed dome with a rose in the centre looks down on the scene of the vanished past. Tradition still asserts that this room was the anatomical theatre, and that the bodies were raised and lowered from a hook to be seen in the middle.

The barbers and surgeons, however, determined on a divorce as in London. In 1739 a petition was presented to the Common Council by the barbers complaining of the impositions and grievances inflicted on them by their surgical brethren. That petition remains unanswered to the present day. The company last appeared in public in 1742, but the surgeons quietly seceded, and the hall was let that year to Mr. Andrew Hooke, and became the West India Coffee House, a well-known resort of the eighteenth century. Ten years later the last surgical member, Mr. Rosewell, died in All Saints' Lane, April 18th, 1752. In place of the old teaching Mr. John Page, a leading surgeon, and Mr. J. Ford started a private course of anatomy in 1744, and again in 1746. Now that the rule of the Company was at an end, the name of barber-surgeon was used by anyone. Hence, probably, much of the contempt with which they were spoken of in later days.

Another portal to medicine arose from the licensing power vested in the bishop of each diocese by the Acts 3 Hen. VIII and 14 and 15 Hen. VIII, which also appointed medical assessors (four in London) to examine the candidates before the bishop granted his licences. No authorities have so far been able to trace these licensing and disciplinary powers to mediæval times. Taken in conjunction with Henry's Charters to the Physicians and Barber-Surgeons, these Acts possibly point to a great scheme which he formed for the complete organisation and reform of medicine, long before he attempted to re-organize the Church. The bishops had the machinery at hand in their visitations, which no other persons had, for searching out irregular practitioners in every parish. Their powers were not a dead letter, for in more than sixty visitations in the seventeenth century[1] I found that inquiries were ordered as to what ignorant persons without a licence from a University or the Ordinary had taken on themselves to practise medicine or surgery. Thus Abbott in 1612 asks how many physicians,

[1] Blue Book, 1868 : Appendix on Injunctions, Visitation articles.

chirurgeons, or midwives there were, and what were their qualifications, skill, and behaviour. Goodman and Laud in 1634 and 1640, and Ironside in 1662, made similar inquiries, and prosecuted offenders in the Bristol diocese, as Mr. Hockaday pointed out to me. There has been discussion too as to how the powers of the bishop and of the barber-surgeons in London worked together. In Bristol an active vicar-general tried in 1670 to make even the barber-surgeons themselves take out licences as well as irregular practitioners. The Company appealed to the Common Council, and the attempt was stopped.

I have made considerable search to find out how numerous the episcopal licences in Bristol were, but the records are very defective. One licence, quoted by Latimer, grants to John Cooper, as late as 1745, the right to practise medicine, mentioning his long experience and his treatise on diabetes. Another, in the possession of our present Bishop, grants to an apothecary, Edmund Tucker, in 1672, a licence in medicine and surgery. There is a licence also to John Hall, of Gloucester, in 1634 for surgery, and a petition from J. Craighton for a similar one. Possibly,where an active Barber-Surgeons' Company existed the demand for the bishop's licences were less than elsewhere.

Medical attendance on the poor was unorganised. There were indeed two or three London hospitals, and Abbot Feckenham, after thirty years of imprisonment, had founded a hospital at Bath, which with Bellot's and Ralph Allen's later gifts, led to the present Mineral Water Hospital, but elsewhere hardly a hospital existed. The Dispensarian Controversy, which many poets and pamphleteers discussed about 1687, points to the needs of the day. The College of Physicians had voted that its members should give advice gratis to their neighbouring poor in London, and for seven miles around. The poor found the cost of medicines prohibitive, and some of the physicians opened a dispensary where drugs were sold at cost price. Other physicians, disliking the plan, refused to meet the founders in consultation, and the apothecaries refused to call them in, while a section of the latter offered to sell drugs to the poor at a low rate. The law courts finally decided against the physicians' dispensary.

*St. Peter's Hospital.*—In Bristol the cost of maintenance of paupers had become very great, owing to the loss of the cloth-making trade in the Temple Parish, and other causes. John Carey proposed a scheme for a common fund for the whole city, and a single poor-house. This was finally confirmed by an Act of Parliament in 1696. The scheme combined voluntary contributions with rate aid, and on the whole was a striking success. Other places imitated Bristol, and finally Poor Law Unions became universal under Gilbert's Act.[1] The old Bristol Mint and a place called Whitehall were purchased to house the people. Local philanthropists subscribed largely, among whom we find Dr. Edward Tyson, then living in London, and our present general poor-rate was started. The first honorary physician was Dr. Thomas Dover, and after him are many great names such as Randolph, who wrote on the Hotwells, and later on Drs. Woodward, Hardwick, Broughton, Cowles Prichard, Fox, and William Budd. Besides an apothecary, there were honorary and paid surgeons, of whom the earliest were Messrs. John Webb, Sandford, and Deverel. In later times such men as Metford and Nehemiah Duck should be remembered. It is amusing to learn that one quarrel with the governing body related to the right of the staff to make *post mortems* on persons dying in the institution. Two of the staff resigned, but a reasonable agreement seems to have been come to.

A Mrs. Dagg was appointed midwife, but she was to call in a surgeon in difficult cases, and the surgeons were to take any and all cases if they wished, it being laid down that Mrs. Dagg's appointment was only to save them trouble. Ringworm, as now, seems to have taxed the skill of the physicians and surgeons, for Mrs. Case was granted five shillings in 1702 for curing "scruffy heads." Mr. Godfrey, too, had a special fee in 1700 for "curing the broken belly" of a man who had lifted a heavy weight; and in 1703 a minute records

[1] Johnson's *History of St. Peter's Hospital.*

that not more than twelve patients suffering from the King's Evil should be sent under the care of a surgeon to Bath to be touched by Queen Anne. This was one of the last occasions when this treatment was available, as the ceremony ceased when she died. Wiseman tells us that about 100,000 persons were touched by Charles II, which cost that impecunious monarch some £10,000 a year.

Dr. Thomas Dover, born 1663, the son of a Warwickshire squire, is one of the most striking characters in Bristol towards the end of the century. He graduated at Oxford and migrated to Cambridge, where he took his M.B. in 1687, after living for a while with Sydenham as pupil. During his pupilage he had the small-pox. Sydenham bled him, and kept him in a room with open windows and no bedclothes higher than the waist, as his custom was.

When recently qualified Dover came to Bristol, where, as we have seen, he was made honorary physician to the new St. Peter's Hospital. He says, "For the encouragement of that good and charitable undertaking I engaged myself to find them physic and to give them advice at my own expense and trouble for the first two years." Typhus or typhoid fever was then raging, and he adds that he visited twenty-five to thirty patients a day, besides "the 200 children in St. Peter's, who nearly all had it, though only one died." [!] He had the audacity to employ the cold bath treatment for a moribund patient, a grocer's apprentice in Wine Street, keeping the man in for a quarter of an hour, after which he recovered completely.

In 1708 he invested capital in Woodes Rogers's expedition to the southern seas, and went himself as second in command. Dr. Nixon[1] remarks that he made himself, as was his wont, an extremely cantankerous travelling companion. Woodes Rogers said his temper was so violent that capable men could not well act under him. However, they came back with enormous gains, and brought with them Alexander Selkirk. I often think what an amusing party he, Selkirk and Defoe must have

[1] *Bristol M.-Chir. J.*, 1909, xxvii, 31.

made at one of the Castle Street inns. Defoe was by turns a hosier, brickmaker, and politician, the most voluminous journalist and novelist in history, a little dark-complexioned man who had been out in Monmouth's Rebellion, the best story-teller in Europe, mobbed in Edinburgh by a home rule crowd, pilloried, and in fact the Mr. Stead of the time.

One of Dover's favourite remedies for various disorders, such gout and asthma, was crude mercury, even an ounce a day for years together. Hence in derision he was called the quicksilver doctor. His fame to-day rests on his celebrated powder, which is used as much as ever. He cherished warmly the memory and teaching of his master Sydenham, and showed considerable knowledge of his art. It is curious to find him quoting Bacon side by side with unctuous letters from grateful patients and furious diatribes against his fellow-physicians and apothecaries, whom he accused of combining to order useless drugs so that the apothecary might run up a bill of £40 or £50 for a single fever.[1]

I have said that a brilliant development of the medical sciences took place in the seventeenth century, and I want to point out the remarkable share taken in it by men of this district. Some five or six different lines of research and possible advance had appeared as early as the Renaissance, and were now bearing fruit, but the time for co-ordination was not yet.

The chief lines of research were :—

(1) The study of Greek medicine and literature, which after being a closed book for centuries, was suddenly thrown open, and led to a great advance in the therapeutic art.

(2) Traditional anatomy was discredited and a new science built on actual observations was steadily developed.

(3) Chemistry, too, sprang into existence, and as it grew was found capable of explaining many things in physiology and medicine.

(4) The astonishing advance in physics and mathematics led many thinkers to believe that the best way to attack

[1] *The Antient Physician's Legacy to his Country.*

physiological and medical problems was through the laws of mechanics. Hence the mechanical theories of physiology and pathology.

(5) The discovery of America gave an impetus to the study of botany and the investigation of many new drugs, such as quinine and guaiacum, as possible specifics.

(6) Lastly the study of clinical medicine, the minute observation of symptoms and the collection of the histories of diseases commended itself to a group of able men as the best method, since the age was clearly not ripe for many applications of the physical sciences.

Let us consider these schools separately, and first the influence of classical and especially of Greek studies. It is curious to note how early local men had come to the front in this. One of the very earliest Grecians was John Free, the son of Bristol parents, who took his degree at Balliol in 1449. He was presented with the rectory of St. Michael's in Bristol, *i.e.* possibly the greater tithes, but the Bishop of Ely sent him and his friend Gunthorpe, like two Rhodes scholars, to study in Italy. He became finally a great teacher of medicine at Florence, Padua and Ferrara in succession, where crowds of picked students from the rest of Europe were gathered. Very few of his writings have come down to us, but it is known that besides his lectures he translated a treatise by Synesius, and at length Pope Paul II, in recognition of his brilliant talents, gave him the bishopric of Bath and Wells, which he did not live to occupy.

Another local man to whom the revival of Greek learning was largely due was the celebrated Grocyn, said to be a Bristolian, but certainly born at Colerne, near Corsham. When Fellow of Merton he was the first teacher of Greek at Oxford, and in 1488 went to Italy for two years and studied, along with Linacre and W. Latimer, under Chalcondyles. So many of the leaders in medicine and theology were trained under him at Oxford that Erasmus speaks of him as the patron and preceptor of us all.

I need not describe how the revival of Greek medicine was carried out by Linacre, Caius, Clement, Baillot, Wotton and the other early members of the College of Physicians. One of the group, Dr. George Owen, the royal physician in three reigns, who is credited rightly or wrongly with performing Cæsarean section at the birth of Edward VI, was either a Bristolian, or in some way connected with the city, to which he became a great benefactor.[1] Henry had given him the spoils of St. Catherine's Hospital in Bedminster, but Owen, when it was safe to do so after the king's death, gave back these lands to the poor of the city, and they now yield £1,500 a year to the Trinity Almshouse.

In the seventeenth century a knowledge of Hippocratic medicine and of classical literature had become the property of the best physicians of every school. Among local men we find Robert Welstead, an M.D. of Oxford and Fellow of the Royal Society, practising at Bristol. He translated Longinus, and wrote various treatises, such as the *De medicina mentis* and *De adultâ ætate*. Musgrave, of Exeter, perhaps the greatest antiquarian of the age, was also Fellow and Secretary of the Royal Society, and wrote various works on joint diseases as well as the *Antiquitates Belgicæ*.

*The Anatomists.*—During the Renaissance period the revived anatomy led to the discovery of new worlds in the microcosm of the human body almost as important as the finding of the new world of America. Vesalius, says Withington, saw that it was useless to go on correcting Galen, and that anatomy must begin afresh. Varolius, Eustachius, Fallopius and Fabricius rapidly raised the new science to importance in Italy. Servetus and Vesalius did the same in France. Vicary popularised the study in England, and regular dissections became the rule, but the immortal Harvey and many others went direct to Padua to drink at the fountain-head. After Harvey's *Anatomical Exercise on the Motion of the Heart and Blood in Animals*, 1628, a crowd of Englishmen carried on the work, such as Cowper, Havers and Wharton.

I would ask your attention, however, to a little group of men from this district who were prominent in research. First let

[1] Author of *A meet diet for the New Ague*, 1558.

us recall G. Joyliffe, a Dorsetshire man, one of the co-discoverers of the lymphatics, together with Bartholini and Rudbeck, in 1651. He announced at once to Glisson what he had found, but did not publish anything on it himself.

Francis Glisson was also a Dorsetshire man, and his grandfather Walter a citizen of Bristol. This *Omnium anatomicorum exactissimus*, as Boerhave called him, became Reader in Anatomy and Regius Professor in Cambridge, and was the first English writer, as Benjamin Richardson remarks, to give a complete account of a particular disease, *i.e.* an account both anatomical and clinical. His *Treatise on Rickets* appeared in 1650. "The precision of his statements shows that each rests on carefully noted observations." Indeed the subject-matter had been threshed out in a medical society before Glisson put it together. He was the first to give an exact proof that when a muscle contracts it does not increase in bulk, and he originated the doctrine of irritability, *i.e.* that the power of responding to stimuli is the special characteristic of living tissue. His doctrine was developed by Haller and Cullen, and caricatured finally by Brown in his theory of excitability, and the value of stimulants, alcoholic and otherwise, for which I must refer to Brown's life by Dr. Beddoes.

W. Charlton, another anatomist, born at Shepton Mallet, followed Charles II into exile with the great surgeon Wiseman, and came back to be President of the College of Physicians. Christopher Bennet, of Wrington, laid the foundation of the pathology of phthisis by his *post-mortem* studies. His great work, *Theatri Tabidorum Vestibulum*, was the received text-book of the time. Edward Tyson, born at Clevedon or Bristol, and one of the founders of St. Peter's Hospital, in 1687, practised chiefly in London, and was a prominent Fellow of the College. He may be called one of the first comparative anatomists, from his monographs on the embryo shark, the tapeworm, the chimpanzee and the rattlesnake. One more great anatomist from this neighbourhood must be mentioned, Thomas Willis born at Great Bedwin, in Wiltshire. He bore arms for the king, and then settled down again at Oxford to practise in a small way at first, attending Abingdon market for patients. Finally he became Sadlerian Professor. In 1661 he moved to London, and obtained a great practice and high honour as one of the greatest physicians and scientists of the time. "Anatomy, physiology, chemistry and the microscope were all now in a state to be serviceable, and Willis employed them all in his medical studies." His labours and discoveries in the physiological anatomy of the brain, cord, and nerves were enormous. The circle of Willis and his classification of the cerebral nerves among other things remain to-day as he and his collaborators left them. He was a chemist as well, and we must now turn back to the founders of—

*The Chemical School.*—Here another line of research had been developed, at first surrounded with occult theories and quackery, but leading to one of the most important and realistic branches of science. Basil Valentine in the early Renaissance had discovered ether, bismuth, and hydrochloric acid; and Paracelsus had an undoubted knowledge of chemistry which he mixed up with various occult doctrines. We find, too, Thomas Norton, of Bristol, like his father, Member of Parliament for the city in 1463, the author of *De Transmutatione Metallorum.* His descendant, Samuel Norton, of St. John's College, Cambridge, and Sheriff of Somerset, lived at Abbot's Leigh, and wrote several treatises on chemistry, such as *The Catholicon Physicorum.* Abroad Van Helmont, the discoverer of $CO_2$, showed that many of the problems of the body are chemical ones, and many of the processes going on in it are true fermentations, like the production of alcohol by yeast. Sylvius, a thorough-going chemist, referred every process, animal heat and cardiac energy included, to effervescence or a fermentation caused by the mingling of acid and alkaline fluids in the body, and disease to an excess of acid or alkali. In England even greater and more solid progress had been made. Lower, a Cornishman, showed that the difference between arterial and venous blood was due to the former taking up fresh air in the lungs; while Mayow, who spent part of his short life at Bath, showed that it takes up only the nitro-aerial part of the air,which supports combustion in the form of a fermentation in every tissue, and is essential for every process and change. This, of course, was an anticipation of the discovery of oxygen.[1]

Willis held similar views, teaching that the blood undergoes a slow burning or combustion, kindled by the air in the lungs, and conveyed over the whole body by the engine of the heart, but that the processes in the organism are forms of fermentation, *i.e.* " an internal motion of the particles of a body leading either to its perfection or change into another form, or what we call metabolism." Disease depends on abnormal fermentations, and the physician is like a brewer whose business is to watch over the process and to correct irregularities.[2]

These doctrines led to wide controversy. Among those who entered into the fray was a Bristol physician, Edward O'Meara, the author of the *Examen Diatribæ Thomæ Willisii*, where he criticises in detail Willis' theory of fevers and some of Harvey's work. He printed with this in 1665 a number of histories of cases of Bristol people and others who came to consult him at the *Aquæ Calidæ* or Hotwells, which we have so utterly neglected to-day to our own loss and that of the city. Francis Cross, of Bristol, whom I have already mentioned, also wrote on the subject, and after taking a medical degree at Leyden practised here " with good success among the Precise party," as Antony a'Wood remarks.

The chemists, not satisfied with annexing physiology, turned their attention to pharmacy and the analysis of mineral waters. Thus we find among local names John Maplet, Student of Christchurch and a friend of Lord Falkland, who practised in the winter at the Hotwells and in the summer at Bath ; Tobie Venner, the author of a *Censure Concerning the Water of St. Vincent's Rocks ;* J. Guidott, the author of *De Thermis Britannicis*, 1681 ; R. Pierce, of Bath ; and later on Caleb Parry, Falconer, and Randolph.

*The Mechanical School* of physiology had numerous

[1] Michael Foster, *History of Physiology*, 1901.

[2] Withington, *Medical History from the earliest times*, 1894.

followers in England at the time. The marvellous work of Newton, Galileo and Boyle had caused mechanical science to advance by leaps and bounds. Hence the recourse to the laws of statics and hydraulics. One would like to dwell on the picture of Sanctorius, surrounded with his new clinical thermometers and instruments of precision, living on his weighing platform, which showed not only the amount of food taken in and excretion, but even the great weight of insensible perspiration. His countryman Borelli, too, for the first time calculated mathematically the forces of muscular contraction, and even the amount of work done by the heart itself. All these forces, he argued, are due to explosions, which are like wedges inserted into the muscles. He even proved that the flow from the arteries to the veins is due to the elastic recoil of the former. Digestion is due to pressure and heat only. Secretion depends on the size of the molecules and the orifices of the sieves through which they have to pass. Pyrexia is not due to acid or alkaline reactions, but to a more rapid flow of the blood, caused by the blocking of certain openings by a viscid fluid. Crude as many of his ideas seem to us, the advance of physiology was enormous, as Michael Foster remarks, and English writers like Pitcairn carried on his method with effect.

William Cole, to whom Sydenham's treatise on small-pox was addressed, was one of the most able of these, and is said by Baas to have lived at Bristol for some time. He wrote on animal secretion and the mechanics of intestinal peristalsis, and advanced a curious explanation of intermittent fevers as being caused by the temporary deposit of abnormal material on the roots of the nerves.[1] We should call this a stimulation of the heat centre by local irritants. Perhaps his greatest contribution is his discovery of the gradual expansion of the arterial tree, from the aorta to the capillaries.

We see, then, that two great attempts were made at this time

[1] William Cole, "Novæ Hypotheseos ad explicanda Febrium Intermittentium symptomata," and "De Mechanicâ ratione Peristaltici Intestinorum motus" 1676.

to solve the problems of disease on purely material lines, the chemical and mathematical. The wonderful group of scientists who founded the Royal Society raised the hopes of men that on these bases a new and firm foundation for medicine might be laid, but their science was not yet perfect nor their instruments complete. Still, the dawn of modern realism, the birthplace of the exact method is seen in this brilliant development of natural science and medicine in England in the seventeenth century.

*The Clinical School* was in a sense the revival of the Hippocratic method. Exact and minnte observation and faithful records of cases had been attempted by a few men, such as perhaps John of Mirfield; Hall, the son-in-law of Shakespeare; and above all Mayerne, who has left us minute details of the fatal typhoid of the Prince of Wales in 1612, and of the physical condition of James I. Twenty-three volumes of his notes have been preserved to this day.[1] But the first to seek general laws as to the course and treatment of disease from clinical observations was Sydenham. He rarely refers to the opinions of others except to those of Hippocrates, but aims at (1) a collection of histories (to use Bacon's phrase) or descriptions of diseases as exact as those in botany, separating them into their several species; (2) a fixed method of treatment founded on experience; (3) a search for specific remedies, which he thinks are probably numerous. A reaction from anatomy and allied sciences was to be expected, for it was clear that they could not yet explain the whole of disease or give the power to control it. Sydenham complains of those "who pompously and speciously prosecute the promoting of this art [of medicine] by searching into the bowels of dead and living creatures." It is true that in acute diseases, which Sydenham chiefly wrote on, there is something which ordinary anatomy will not explain, yet, as Payne says, we now admit this specific factor and call it a microbe. But in organic diseases, in dropsy and disturbances of the circulation, anatomy and physiology are all-important. Again, it might be said that for a cavalry

[1] Norman Moore, *History of the study of Medicine in the British Isles*, 1908.

officer like Sydenham, turned doctor, the drudgery of chemistry and anatomy would be specially distasteful. Indeed, the tendency had been always great for physicians and surgeons to throw over the rougher parts of their work to apothecaries and others, and I hear someone remark that the same thing exists among us to-day. However, if he, like his contemporaries, was one-sided, the lesson most needed by the age was the necessity of observing and recording facts as they really are, of avoiding hypotheses based on theories instead of facts, and of giving the healing power of Nature full play under careful management. Many of the best men of the day developed his method.

I have not time to sketch the life of Sydenham himself. Suffice it to say that he came of a Somerset family, members of which fought on either side in the Rebellion. Born in Dorsetshire, he fought long and well for the Parliament, his mother and two brothers were killed in the war, and his eldest brother was made Governor of Bristol. No man probably from this district has influenced European medicine so much as he has done.

One more local worthy must be mentioned, a warm friend and follower of Sydenham, the immortal John Locke, best known for his essay on the "Human Understanding," but also a practitioner of physic. His father practised here at Pensford as an attorney, and John was born in 1632, while his mother was on a visit to her brother at Wrington. He went to Westminster and Christchurch, and had an ample income from his Fellowship and his estate in Somerset, so that he died possessed of £45,000. He became an assistant to an Oxford practitioner, and then lived for years as medical adviser to Lord Ashley, on whom he operated for a hydatid cyst of the liver. This he opened and drained by a silver tube, which, being kept in permanently, became the talk of the day. Many notes of his other cases have survived. He wrote, too, on Sanitary and Poor-Law Reform, and was for some time Commissioner of Trade and Plantations. It is curious to find him, like Sydenham, advocating open-air exercise in phthisis.

In conclusion, you will ask what is the use of all this story of the past, this raking-up of the anatomies of dead men. First, I think, we see what gigantic problems may be solved by persistent effort. We, too, have wide fields to explore—cancer, apoplexy, neural and renal disease, for which we can do very little; but the men of the seventeenth century, with fewer resources, overcame even greater difficulties. Next we realise that medical education and practice must be organised. Even now vast masses of men, as Mr. Lloyd George tells us, are practically without medical care, and as to the rest no organised register of each man's diseases through life, not to speak of his family history, exists as a rule. We are left to the patient's vague recollections. Finally, we learn that though medicine rests on the subsidiary sciences, it is more than one or all of them. One-sided specialism did not then and cannot now produce a therapeutic art of any value to mankind.

